# Coronary Microvascular Dysfunction: PET, CMR and CT Assessment

**DOI:** 10.3390/jcm10091848

**Published:** 2021-04-23

**Authors:** Elisabetta Tonet, Graziella Pompei, Evelina Faragasso, Alberto Cossu, Rita Pavasini, Giulia Passarini, Matteo Tebaldi, Gianluca Campo

**Affiliations:** 1 Cardiovascular Institute, Azienda Ospedaliero-Universitaria di Ferrara, 44124 Cona, Italy; graziella.pompei@outlook.it (G.P.); pvsrti@unife.it (R.P.); giulia.passarini.gp@gmail.com (G.P.); tblmtt@unife.it (M.T.); cmpglc@unife.it (G.C.); 2 Department of Cardiology and Cardiac Surgery, Cardiovascular Imaging, GVM Care&Research, 42121 Reggio Emilia, Italy; evelina.faragasso@gmail.com; 3 Department of Morphology, Surgery and Experimental Medicine, Section of Radiology, University of Ferrara, 44124 Ferrara, Italy; csslrt@unife.it

**Keywords:** microvascular dysfunction, angina, positron emission tomography, cardiac magnetic resonance, cardiac computer tomography

## Abstract

Microvascular dysfunction is responsible for chest pain in various kinds of patients, including those with obstructive coronary artery disease and persistent symptoms despite revascularization, or those with myocardial disease without coronary stenosis. Its diagnosis can be performed with an advanced imaging technique such as positron emission tomography, which represents the gold standard for diagnosing microvascular abnormalities. In recent years, cardiovascular magnetic resonance and cardiac computed tomography have demonstrated to be emerging modalities for microcirculation assessment. The identification of microvascular disease represents a fundamental step in the characterization of patients with chest pain and no epicardial coronary disease: its identification is important to manage medical strategies and improve prognosis. The present overview summarizes the main techniques and current evidence of these advanced imaging strategies in assessing microvascular dysfunction and, if present, their relationship with invasive evaluation.

## 1. Introduction

Chest pain without obstructive coronary disease (CAD) represents a frequent phenomenon in clinical practice. Patel MR et al. demonstrated that nearly 60% of symptomatic patients undergoing invasive diagnostic coronary angiography did not show obstructive CAD [1]. Currently, the rate of these patients varies but it is about 15% [2]. Invasive flow assessment suggested that up to two-thirds of the patients have microvascular dysfunction (MVD). The high rate of MVD has made it of paramount importance to understand the physiology of microcirculation. Furthermore, it has been demonstrated that the degree of microvascular impairment carries important prognostic relevance. Previous studies showed that MVD had a predictive value in terms of cardiac death in various populations, such as in women, patients with diabetes mellitus, chronic kidney disease, hypertrophic obstructive cardiomyopathy, ischemic or idiopathic cardiomyopathy [3,4].

**Figure 1:**
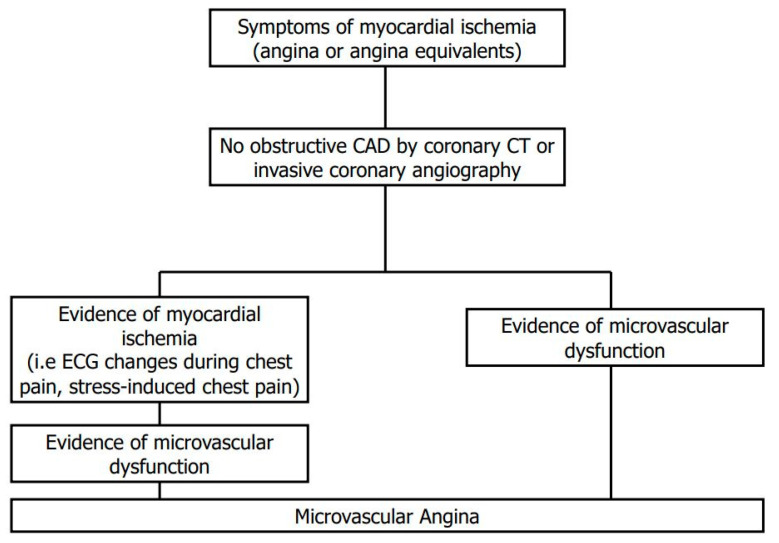
Flow chart for the diagnosis of microvascular angina according to current criteria. CAD = coronary artery disease; CT = computed tomography.

As a result, assessing microvascular function would represent a fundamental step in evaluating the increasing population with angina without obstructive CAD (Figure 1) [5]. To date, the development of sophisticated imaging techniques allows the assessment of MVD without any complication related to invasive strategies. This review aims to summarize the main concepts about the physiology of microcirculation and highlight the current evidence on established and emerging imaging techniques, namely positron emission tomography (PET), cardiovascular magnetic resonance (CMR) and cardiac computed tomography (cardiac CT). 

## 2. Microvascular Physiology and Dysfunction

**Figure 2:**
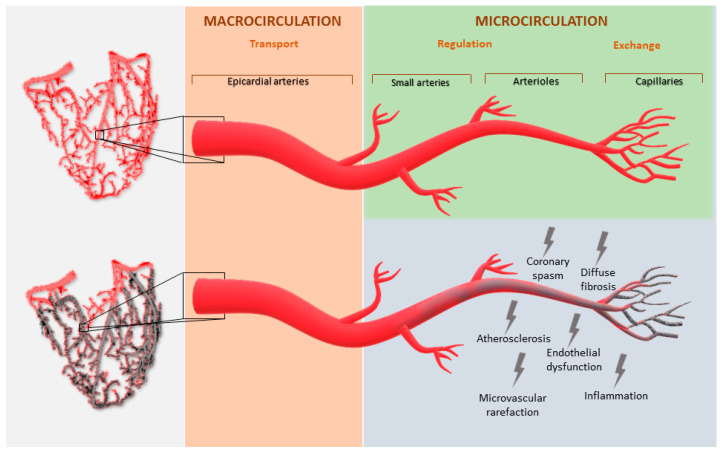
Mechanisms of microvascular dysfunction.

Myocardial perfusion is governed by dynamic and combined changes in the epicardial coronary vessels and microcirculation. The three components of the coronary arterial vasculature are epicardial coronary arteries, pre-arteriole vessels and the intramural arterioles. The last two have the key role of matching blood supply to myocardial oxygen consumption. At rest, the myocardium extracts 75% of the blood oxygen and any increase in oxygen consumption determines increased oxygen demand, which leads to an increase in myocardial blood flow (MBF) [5]. MBF is mainly regulated by microcirculation, and it can be defined as the amount of flow through the coronary vessels expressed as blood flow per gram of myocardium [5,6]. Pre-arteriole vessels and arterioles regulate MBF with various mechanisms including arterial tone and diameter. In the setting of MVD, there is a disruption of these mechanisms due to several factors including endothelial dysfunction, coronary spasm, inflammation, atherosclerosis, microvascular rarefaction, and diffuse fibrosis (Figure 2) [7,8,9]. These abnormalities determine alterations in the blood supply distribution and consequent chest pain.

MVD can be recognized in three different settings. First, MVD without obstructive CAD and myocardial diseases due to cardiovascular risk factors [10]. These patients could be asymptomatic, but they represent a population with a higher risk of CAD development. Second, MVD with obstructive CAD. Many patients with stable CAD and acute coronary syndrome also show MVD. On the one hand, it plays a key role after stenting implantation in patients with stable CAD who remain symptomatic. On the other hand, in acute coronary syndrome, MVD is responsible for the no-reflow phenomenon after revascularization [11]. Third, MVD in myocardial diseases. MVD can be identified in patients suffering from hypertrophic or dilated cardiomyopathy or structural myocardial abnormalities due to severe valvular diseases, such as aortic stenosis [12]. 

Regardless of the mechanism behind MVD, it could be non-invasively assessed. Table 1 shows the mechanisms of MVD in the above-mentioned clinical scenarios, and for each one, it suggests the most appropriate imaging techniques. 

## 3. Cardiac PET

Cardiac Positron Emission Tomography (PET) represents the most validated imaging exam for the non-invasive identification of MVD. It is based on the use of tracers labeled with isotope emitting positrons; it has high sensitivity and temporal resolution, which allows fast dynamic imaging of tracer kinetics [13]. The ideal radiotracer would be completely safe, without side effects, freely diffusible, and it would have a high first-pass uptake, rapid clearance rate and kinetics not influenced by extrinsic factors [13]. The main characteristics of PET tracers are summarized in Table 2. The American Society of Nuclear Cardiology and the Society of Nuclear Medicine and Molecular Imaging recommend the use of myocardial perfusion PET in clinical practice because of its properties: high diagnostic accuracy, consistent high-quality images, short acquisition protocols, strong prognostic power, and low radiation exposure [14].

The use of rest and stress PET allows the quantification of some indices of MVD, such as MBF, myocardial perfusion reserve (MPR: MBF at the maximum stress) and myocardial flow reserve (MFR: ratio of MBF during maximal coronary vasodilatation to resting MBF) [14]. In particular, an MFR < 1.5 suggests a reduced flow reserve and MVD [15]. MVD assessment with PET has been evaluated in various settings and demonstrated a prognostic value in some of them. Quinones et al. showed that insulin-resistant patients have impaired microvascular vasomotion using PET at rest, during the cold pressor test (endothelium-dependent), and after dipyridamole administration (vascular smooth muscle-dependent). On average, myocardial blood flow responses to the cold pressor test were reduced by 70% of that observed in insulin-sensitive patients. This abnormal response occurred despite a normal flow response to dipyridamole, suggesting a potential abnormality of the coronary endothelium [16]. Previous studies also reported abnormal PET-derived MBF in patients with metabolic syndrome and non-insulin-dependent diabetes [17,18]. Taqueti et al. demonstrated that women frequently showed impaired flow reserve assessed by PET, without obstructive CAD; this population also had a significantly increased adjusted risk of CVD events (*p* < 0.0001, *p* for interaction = 0.04) [19]. In another study, Taqueti VR et al. also demonstrated that abnormal flow reserve in patients without CAD was associated with diastolic dysfunction and a high risk for hospitalization for heart failure [20]. PET was also used to assess MVD in myocardial and valvular diseases such as aortic stenosis and hypertrophic cardiomyopathy (HCM). Regarding the latter, Bravo et al. performed PET in 33 symptomatic HCM patients demonstrating good performance in the quantification of MBF and MFR [21]. Moreover, PET flow quantification has promising potential for the non-invasive evaluation of cardiac allograft vasculopathy after heart transplantation. In this setting, Chich et al. showed a good correlation between 82Rb-PET-determined myocardial flow and invasive coronary flow measures [22]. In STEMI patients, PET was used as a gold standard for comparison with microvascular indexes invasively measured immediately after PCI in terms of predicting left ventricular functional improvement (*r* = 0.442) [23]. Therefore, a good correlation between PET and invasive physiologic indices to evaluate microvascular dysfunction has been established. Furthermore, PET could explain some discrepancies between invasive parameters. Lee et al. investigated patients with discordant values by invasive assessment: using PET evaluation of MVD, they demonstrated that this discrepancy could be due to microvascular function [24]. Although PET is the gold standard for the non-invasive assessment of microvascular function, the use of this technique in clinical practice is still limited due to some factors shown in Table 3. To overcome this problem, a new high-sensitivity 3D PET scanner was developed to reduce radiation dose, maintaining high measurement and quantification quality [25]. 

## 4. Cardiovascular MRI

**Figure 3:**
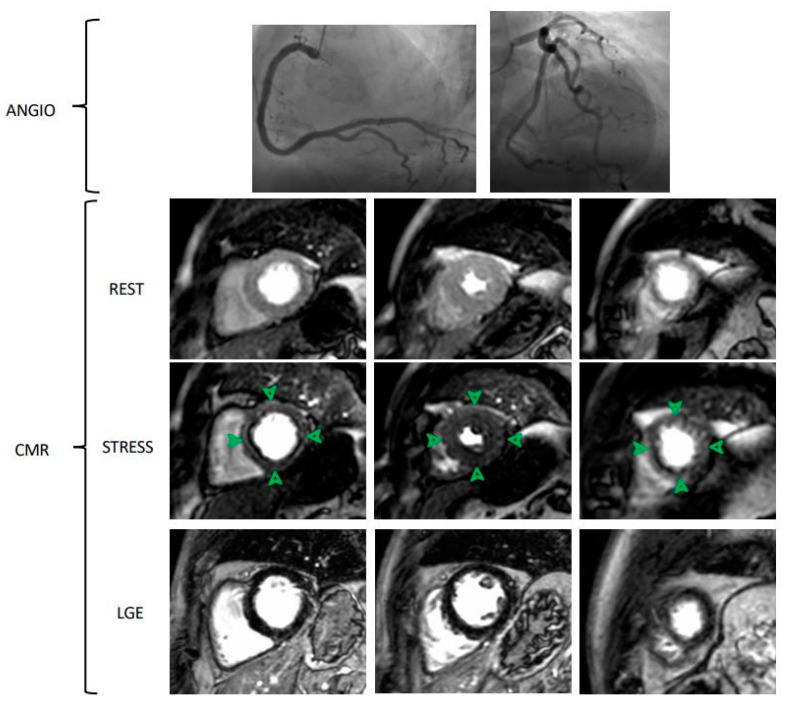
Cardiac Magnetic Resonance (CMR) evaluation of microvascular dysfunction in a patient with effort angina without obstructive coronary artery disease. Invasive coronary angiography shows epicardial coronary artery without obstructive disease. CMR sequences show three ventricular slices (basal, mid-ventricular, apical slices) during rest, stress and late gadolinium enhancement (LGE) protocols. Stress CMR is performed with adenosine administration (140 mcg/Kg/min for 3–6 min). Comparing rest and stress CMR sequences, there is a severe and diffuse hypoperfusion in the stress images, showed by a widely hypointense myocardium (green arrows): this pattern is consistent with microvascular dysfunction. LGE images show no myocardial fibrosis. CMR Sequences: Saturation recovery gradient echo pulse sequences for rest and stress images; Inversion recovery gradient echo sequences for LGE images.

CMR seems to be a promising non-invasive imaging technique in the assessment of myocardial perfusion and flow quantification, given its high spatial resolution, lack of radiation, and good diagnostic accuracy [26]. As previously written about PET, MBF and myocardial perfusion reserve (MPR) can also be calculated by rest and stress perfusion CMR. Visual assessment and semi-quantitative methods for perfusion assessment are routinely used. Conversely, to date, quantitative analysis of MBF with CMR is only performed in a research setting. Briefly, visual assessment is based on capturing the first-pass transit of gadolinium in the myocardium. This is because well-perfused myocardium has shorter T1 relaxation time and so it appears bright, while perfusion deficits appear as an area of lower signal intensity. In the absence of obstructive CAD, these data could be related to impaired MBF and MVD (Figure 3). First-pass imaging can also be used for semi-quantitative assessment of MPR, collecting signal intensity data before and after gadolinium administration. Dividing the results at maximum vasodilatation (i.e., with dipyridamole) by the results at rest, the MPR index is obtained. If the MPR index was less than or equal to 1.5, the myocardial segment was classified as pathological [27]. Larghat Am et al. demonstrated good reproducibility with a low inter- and intra-observer variability [28]. 

However, some limitations should be underlined, such as the MPR index, which is influenced by resting perfusion and by tissue contrast concentration [29]. Quantitative assessment includes compartmental kinetic models and deconvolution methods: the first one provides time signal intensity curves and kinetic models to quantify MBF; the second one estimates MBF, assessing some parameters such as arterial input function, tissue response and intravascular and extravascular tracer concentration. Several previous studies investigated CMR performance in MVD identification. Initial animal models evaluating MBF assessment by CMR demonstrated its good correlation (*r* > 0.90) with microsphere analysis, which represented the gold standard [30,31]. Studies in humans firstly compared MVD assessment by CMR with PET in various clinical settings. In patients with stable CAD, Engblom H et al. showed a good agreement (*r* = 0.92) between CMR and PET in the assessment of global MBF [32]. In a cohort of women with angina and no obstructive CAD, Mygind et al. showed a moderate but significant correlation between CMR and PET in MVD identification (*r* = 0.46, *p* < 0.001) [33]. The results of CMR also correlated well with data from invasive measurements, independently from biomarkers of atherosclerosis [34]. A recent study validated a novel automated inline myocardial perfusion mapping technique for the assessment of MVD. This study demonstrated that MVD defined by the invasive index of microcirculatory resistance was well recognized by CMR and that this technique was able to distinguish between MVD and multivessel epicardial disease [35]. CMR also allows the contrast-free assessment of coronary blood flow with stress T1 mapping [36]: in a cohort of 31 patients with type 2 diabetes mellitus and without significant CAD, Levelt E et al. showed a blunted maximal non-contrast T1 response during stress with adenosine administration, reflecting MVD [37]. Mahmod M et al. investigated microvascular compartment using stress and rest T1 mapping in patients with aortic stenosis and no obstructive CAD demonstrating an elevated resting T1, reflecting microvascular vasodilatation related to pressure overload and hypertrophy [38]. 

**Figure 4:**
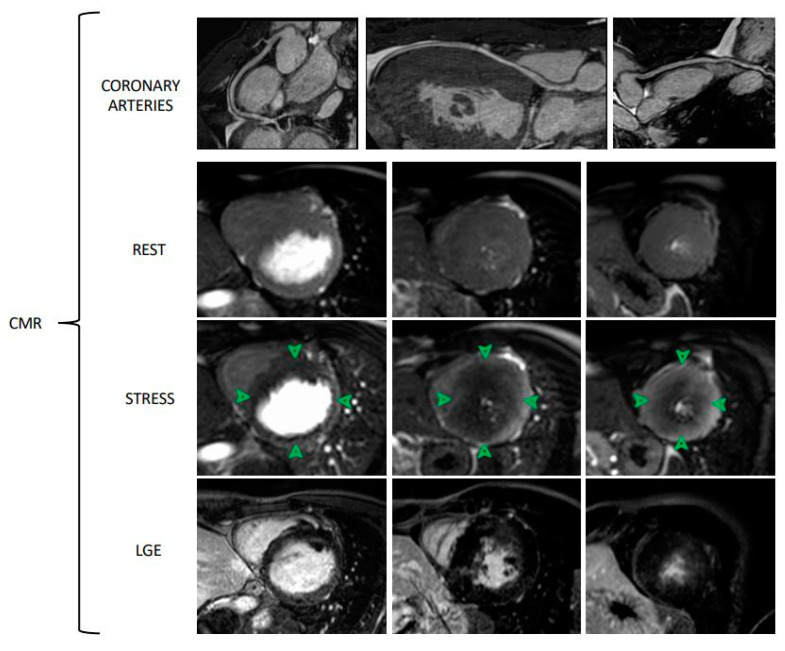
CMR assessment of microvascular dysfunction in a young patient with Danon disease. CMR analysis includes sequences for the visualization of epicardial coronary arteries, rest and stress protocols and LGE images of three ventricular slices (basal, mid-ventricular, apical slices). Stress CMR is performed by adenosine administration (140 mcg/Kg/min for 3–6 min). Comparing rest and stress images, a diffuse subendocardial hypoperfusion (green arrows) due to microvascular dysfunction can be noted. LGE images show no ischemic pattern. CMR Sequences: Free-breathing diaphragmatic 3D navigator BFTE Whole heart non contrast sequences for coronary arteries images, Saturation recovery gradient echo pulse sequences for rest and stress images, and Inversion recovery gradient echo sequences for LGE images.

Recently, the prognostic value of MVD assessment by CMR was investigated. In a cohort of 218 patients with angina and without overt CAD, Zhou W et al. demonstrated that CMR-derived MPR index was an independent predictor of adverse events [39]. Finally, in research settings, CMR was also used for the anatomic assessment of epicardial coronary vessels [40]. Therefore, CMR could potentially become a comprehensive assessment method in patients with angina, without radiation exposure: from the detection of coronary artery disease to the identification of MVD (Figure 4). However, the use of CMR for the assessment of MVD also shows some limitations, such as the time-consuming post-processing. The main advantages and disadvantages of CMR are reported in Table 3.

## 5. Cardiac CT

Cardiac CT has recently been increasingly used for functional testing [41]. CT angiography associated with CT perfusion (CTP) could have good performance in the assessment of microvascular disease. CTP consists of the evaluation of the passage of contrast medium from the vascular to the myocardial compartment at rest and after adenosine administration. The attenuation of radiation by the contrast agent is proportional to its amount: as a result, reduced density areas, either hypo-enhanced or non-enhanced, represent regions with reduced perfusion in the myocardium.

The combination of these two techniques allows obtaining both coronary anatomic and myocardial perfusion information in the same study. CTP requires the acquisition of an ECG gated intravenous iodinated contrast-enhanced CT during vasodilator stress. Scan acquisition is performed during the early first pass of contrast into the myocardium to observe differences in the inflow of contrast and, therefore, differences in attenuation of normal and impaired myocardium [42]. 

Two types of CTP can be identified: static and dynamic.

- Static CTP requires only a single image at peak myocardial contrast opacification, which is then compared with a single rest image. This technique requires prospective ECG triggering and is associated with a lower amount of radiation, but it allows only semiquantitative or qualitative perfusion evaluation;
- Dynamic CTP obtains several sequential images over time from the first pass to the wash-out of contrast medium, allowing the calculation of the kinetics of iodinated contrast in the arterial blood pool and myocardium over time. As a consequence, a quantitative perfusion estimation is obtained. This method is related to quantifying MBF, but it requires a 3-fold higher radiation exposure than static CTP [43].

The major advantages of CT are shown in Table 3. Briefly, the possibility of dynamic CT imaging allows the calculation of intramyocardial blood volume; additionally, the high spatial resolution makes this technique able to distinguish between the endocardium and the epicardium and the attenuation difference between these two layers. This concept is of paramount importance: decreased endocardial over epicardial contrast ratios and the consequent attenuation values were shown to be associated with MVD [44].

However, studies about CTP for the assessment of MVD are still few. In animal models, MBF quantification by CTP correlated well with microsphere measurements and with CMR [45,46]. 

Alessio AM et al. compared dynamic CTP with rubidium-82 PET in MBF estimation in a prospective trial on high-risk patients. The study demonstrated that CT-derived MBF estimates were, on average, equivalent to quantitative PET estimates (*r* = 0.92) [47]. As far as we know, no studies compared CTP with an invasive assessment of microvascular dysfunction. Nevertheless, considering that CT allows an optimal investigation of epicardial coronary artery and microvascular function in the same exam, it could be a promising technique for a comprehensive assessment. 

## 6. Limitations and Conclusions 

Despite the relevant advantages of non-invasive techniques, some limitations should be recognized. First, no stressors are available to assess endothelial function or coronary spasms. Indeed, vasodilators used in non-invasive techniques (i.e., adenosine or dipyridamole) only assess vasodilator capacity. Second, before formulating the diagnosis of angina due to MVD, obstructive CAD has to be excluded. Therefore, it can be concluded that these techniques have a good negative predictive value but a limited positive predictive value. This concept plays a pivotal role in the setting of diffuse three-vessel CAD, which can be misinterpreted as MVD. To date, the only comprehensive non-invasive technique for the ruling-out of epicardial CAD in clinical practice is cardiac CT. In conclusion, according to current data about advanced non-invasive techniques for the identification of MVD, PET, CMR, and CT could be very promising modalities. Recognizing coronary MVD is of paramount importance because of its prognostic role, and the goal is the definition of therapeutic approaches [48].

## Figures and Tables

**Table 1:** Three clinical scenarios of MVD and the suggested assessment modalities.

Clinical Scenario	Patophysiology	PET	CMR	CT
MVD without obstructive CAD or myocardial diseases	Cardiovascular risk factors, such as hypertension and diabetes, determine endothelial dysfunction and abnormal function of vascular smooth muscle cells.	The most tested in this setting with a prognostic role	Tested in this setting	Contemporary assessment of epicardial vessels and MVD
MVD in the presence of obstructive CAD	Stable CAD: atherosclerotic involvement of microcirculation and endothelial dysfunction. Acute coronary syndrome: microvascular obstruction due to edema, hemorrhage and inflammation.	Tested in this setting	Tested in this setting and useful for tissue characterization	Contemporary assessment of epicardial vessels and MVD
MVD in the presence of myocardial or severe valvular diseases	Structural alterations (i.e., hypertrophy or interstitial fibrosis) determine capillary rarefaction and increase arterial stiffness.	The most tested in this setting	Tested in this setting and useful for tissue characterization and valvular diseases estimation	Not tested and limited usefulness in this setting

**Table 2:** Characteristics of the main PET radiotracers.

Radiotracer	Half-Life	Advantages	Disadvantages
^15^O-water	120 s	-High myocardial extraction fraction	-Limited application to facilities with an on-site cyclotron
^82^Rubidium	76 s	-Not requiring a cyclotron on site	-Significant roll-off at high flows -Low myocardial extraction fraction
^13^N-ammonia	10 min	-High myocardial extraction fraction	-Requiring a cyclotron on site
^18^F-labeled agents	variable	-Flow-independent high extraction fraction (>90%)	-Alterations in the metabolic state of the myocardium may affect its retention -Current use only in investigational trials

**Table 3:** Pros and cons of the three advanced imaging techniques.

Modality	Protocol	Pros	Cons
PET	Vasodilator stress and rest perfusion images	-Most validated technique -Prognostic values -Good reproducibility -Not limited by renal function	-High costs -Radiation exposure -Limited availability -Time consuming procedure
CMR	Vasodilator stress and rest perfusion images	-High spatial resolution -Tissue characterization -No radiation -Validated and compared with PET and invasive methods -Anatomic evaluation of epicardial coronary vessels (limited data)	-High costs -Limited by renal function -Limited availability -Poor prognostic data -Time consuming
CT	Vasodilator stress and rest perfusion images	Anatomic and functional data in the same study	-Limited availability -Limited by renal function -Radiation exposure -Risk of MBF overestimation

## Data Availability

Not applicable.
